# Femoral neck width is associated with unique trajectories of age-related hip structural changes and fracture risk within populations of adult women and men

**DOI:** 10.1093/jbmr/zjaf090

**Published:** 2025-06-28

**Authors:** Karl J Jepsen, Todd L Bredbenner, Carrie A Karvonen-Gutierrez, Aleda M Leis, Michelle M Hood, Siobán D Harlow, John Randolph, Gregory A Clines, Steffenie Merillat, Michael R Elliott, Jane A Cauley, Gail A Greendale, Arun S Karlamangla, Katherine W Peters, Stephanie L Harrison, Li-Yung Lui, Peggy M Cawthon, Eric Orwoll

**Affiliations:** University of Michigan, Department of Orthopaedic Surgery, Ann Arbor, MI 48109, United States; University of Colorado Colorado Springs, Department of Mechanical and Aerospace Engineering, Colorado Springs, CO 80918, United States; University of Michigan, Department of Orthopaedic Surgery, Ann Arbor, MI 48109, United States; University of Michigan, Department of Orthopaedic Surgery, Ann Arbor, MI 48109, United States; University of Michigan, Department of Orthopaedic Surgery, Ann Arbor, MI 48109, United States; University of Michigan, Department of Orthopaedic Surgery, Ann Arbor, MI 48109, United States; University of Michigan, Department of Orthopaedic Surgery, Ann Arbor, MI 48109, United States; University of Michigan, Department of Orthopaedic Surgery, Ann Arbor, MI 48109, United States; University of Michigan, Department of Orthopaedic Surgery, Ann Arbor, MI 48109, United States; University of Michigan, Department of Orthopaedic Surgery, Ann Arbor, MI 48109, United States; University of Pittsburgh, Department of Epidemiology, Pittsburgh, PA 15261, United States; University of California, Department of Medicine, Los Angeles, Los Angeles, CA 90095, United States; University of California, Department of Medicine, Los Angeles, Los Angeles, CA 90095, United States; California Pacific Medical Center Research Institute, San Francisco, CA 94158, United States; California Pacific Medical Center Research Institute, San Francisco, CA 94158, United States; California Pacific Medical Center Research Institute, San Francisco, CA 94158, United States; California Pacific Medical Center Research Institute, San Francisco, CA 94158, United States; Oregon Health and Science University, Division of Endocrinology, Diabetes, and Clinical Nutrition, Portland, OR 97239, United States

**Keywords:** aging, external bone size, DXA, hip, fracture, menopause, biomechanics, osteoporosis

## Abstract

Osteoporosis management relies heavily on areal BMD (aBMD) to identify women and men with reduced bone strength. We tested the hypothesis that baseline FN external size is associated with different bone-loss and area-gain trajectories that are not reflected in aBMD-decline but have different biomechanical implications. We analyzed data from 4 longitudinal studies with repeated hip DXA scans of women and men over 10-15 yr of follow-up. Changes in FN BMC, area, and aBMD were compared across height-adjusted baseline FN area tertiles using linear models. Fracture risk differences across the tertiles were tested using Cox proportional-hazard models. Women and men with smaller baseline FN area had smaller BMC-declines and greater area-increases over 10-15 yr. In contrast, those with a larger FN area experienced twice the annual BMC-declines but much smaller area-increases. In general, these structural changes were not reflected in aBMD-changes for either sex. The likelihood of fracturing a hip was 2.5 times greater for women and 2.4-4.2 times greater for men in the larger FN area tertile compared to those in the smaller FN area tertile. Unique patterns of age-related structural changes with different biomechanical implications were identified within populations of women and men. These results challenge the general assumption that age-related structural changes are homogenous within a population and question whether aBMD-declines reflect strength-declines consistently among women and men. How these unique patterns of structural change affect the response of women and men to osteoporosis interventions remains to be determined.

## Graphical Abstract

**Graphical Abstract:**
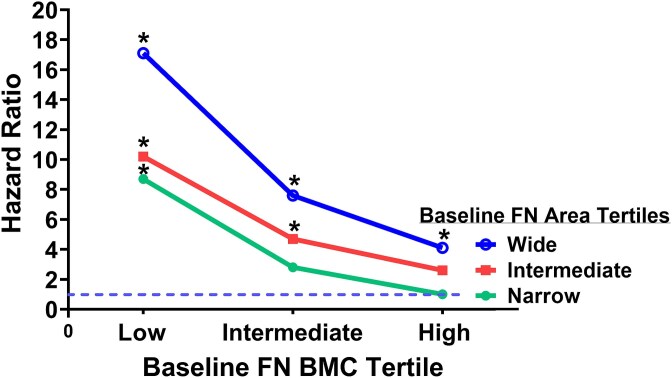

## Introduction

Hip fractures are a leading cause of morbidity and mortality among older adults^1^ and are associated with loss of independence and decline in quality of life.^2^ An age-related increase in fracture risk results largely from a decline in bone strength, which is the maximum load a bone can sustain before breaking. Age-related changes in structure (eg, reduced cortical thickness) and material properties (eg, increased brittleness) can weaken bone to the point when minimal trauma (eg, fall) results in a fracture. Hip areal BMD (aBMD) measured from DXA is commonly used for identifying individuals with reduced bone strength and increased fracture risk. Declines in aBMD are generally thought to reflect declines in strength but changes in aBMD have been an inconsistent predictor of fracture risk.^3–5^ The expected worldwide increase in hip fractures^6^ and the use of changes in aBMD to assess treatment efficacy^7^ or identify those most likely to benefit from treatment further underscore the importance of understanding whether bone health measures like aBMD adequately reflect bone strength across a population.

Femoral neck aBMD is calculated as the ratio of BMC to the projected area of the FN region. Loss of aBMD is generally thought to reflect a loss of BMC, but less attention has been given to how changes in area also affect aBMD.^8–12^ External bone size increases with aging through periosteal apposition leading to increases in FN area.^13^ It is generally thought that increases in outer bone size mechanically offset the effects of age-related bone loss on strength. Importantly, the amount of bone loss relative to area gain affects how well a reduction in aBMD reflects loss of strength. This is because both BMC-declines and area-increases lead to reduced aBMD, but they have opposite effects on bone strength.^13^^,^^14^ In our prior work, we reported population subgroups showing different patterns of biomechanically-important baseline structures and structural changes over time that were not detected by changes in aBMD.^11^ The relative changes in FN BMC and area differed among menopausal women, with baseline FN area being associated with heterogenous trajectories of BMC-change and area-change over 14 yr of follow-up.^11^ More specifically, women with a narrower FN at baseline (ie, smaller FN area) showed small decreases in BMC but large increases in area, whereas women with a wider FN at baseline (ie, larger FN area) showed large reductions in BMC but only small increases in area. The different relative changes in BMC and area are expected to lead to minimal declines in strength for narrower FNs but large declines in strength for wider FNs, consistent with prior work.^15^^,^^16^ Critically, the different trajectories of BMC and area change in those with narrower vs wider FNs resulted in similar magnitudes of aBMD loss.

Overall, our previous results have several potentially important clinical implications. First, these findings challenge the general assumption that the trajectories of age-related structural changes are similar among individuals.^17^ Second, the fact that greater BMC declines did not correspond to greater area increases suggests further investigation is needed to understand the association between periosteal expansion and age-related bone loss. Finally, our findings indicated that the underlying causes of aBMD-decline vary among individuals and that aBMD does not adequately distinguish among those aBMD-decline mechanisms, a critical concern since those different aBMD-decline mechanisms may have quite different biomechanical implications. Together, our findings have called into question whether declines in aBMD adequately reflect loss of bone strength.

We believe the ability to identify novel fracture-related metrics has been limited by not recognizing the variation of age-related structural changes, nor acknowledging that aBMD may not reflect this variation. Herein, we replicate our prior study, which was limited to a small cohort of women undergoing the menopause transition,^11^ in larger cohorts of women experiencing the menopause transition and expand the sample to include older postmenopausal women and older men. The current study had a set of 3 hypotheses. First, we tested the hypothesis that baseline FN area measured from routine hip DXA scans is associated with different annual losses of FN BMC and annual increases in FN area. Second, we tested if these different patterns of structural change were consistently reflected in annual loss of FN aBMD. Third, we tested if baseline FN area was associated with hip fracture risk in older women and men.

## Materials and methods

### Study samples

**Figure 1:**
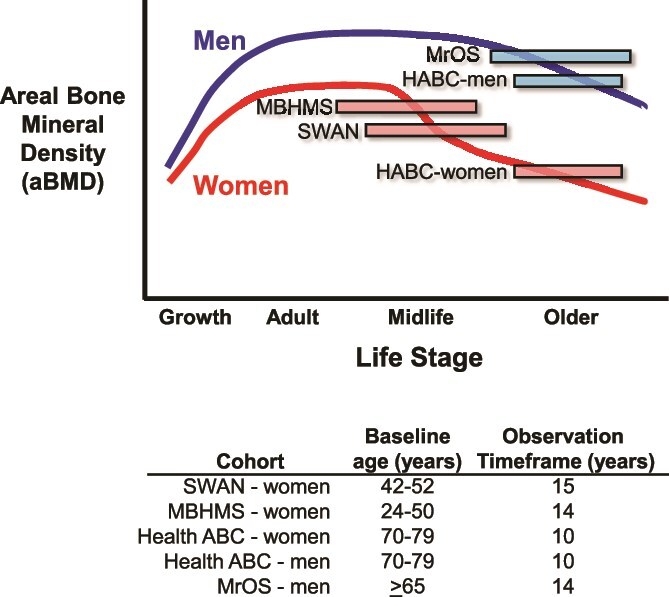
Schematic showing baseline ages and observation timeframes relative to age-changes in FN aBMD for the 4 longitudinal cohorts used in the analysis. Age changes in aBMD are shown for schematic purposes and are consistent with previous reports.^51^

Four prospective longitudinal observational studies were analyzed. Together, these studies enrolled over 13 000 women and men. They included the Study of Women’s Health Across the Nation (SWAN), the Michigan Bone Health and Metabolism Study (MBHMS), the Health, Aging, and Body Composition Study (Health ABC), and the Osteoporotic Fractures in Men Study (MrOS). The longitudinal observation periods spanned 10-15 yr across ages when important age-related structural and strength changes occur, including among midlife women (SWAN, MBHMS), older women (Health ABC-women), and older men (Health ABC-men, MrOS) (Figure 1). Because the rate of bone loss differs between the menopausal transition and postmenopausal stages of reproductive life for women,^9^ longitudinal cohorts of women spanning the midlife and older age were included to test if external-size dependent patterns of bone changes are observed for both timeframes. Men enrolled in Health ABC and MrOS were included to test if the external-size dependent patterns of bone changes reported previously for women^11^ are also observed for men. Written informed consent was obtained from all participants of all cohorts and at each study visit. In all cohorts, height was measured using stadiometers and weight was measured with a calibrated balance beam or digital scale. Race/ethnicity was self-reported. Approval for the conduct of the studies was obtained from each site’s Institutional Review Board.

The Study of Women’s Health Across the Nation (SWAN) is a multi-site, prospective cohort study of women transitioning through menopause. SWAN enrolled 3302 women at 7 geographically and ethnically diverse centers in the United States. The cohort includes White (47%), Black (28%), Japanese (9%), Chinese (8%), and Hispanic (9%) women.^18^ Five of the seven centers (Boston, MA; Detroit-area, MI; Los Angeles, CA; Pittsburgh, PA; Oakland, CA) obtained DXA data on participants with approval of each site’s institutional review boards. Study details have been described previously.^19^ Briefly, eligibility criterion included 42-52 yr of age at baseline in 1996, having an intact uterus, at least 1 menstrual period in the previous 3 mo, and no use of exogenous hormones in the previous 3 mo. All women were pre- or early perimenopausal at baseline, whereas at the 15-yr follow-up visit, nearly all women were postmenopausal.

The MBHMS is a population-based longitudinal observational study of ovarian aging and musculoskeletal health in women during their mid-adulthood.^20^ MBHMS began in 1992 when women were aged 24-50 yr and included 664 White women who were followed for 14 yr. DXA data was obtained on non-pregnant participants at each study with the approval of the University of Michigan Institutional Review Board.

The Health ABC is a multi-site, prospective cohort study designed to examine risk factors associated with the decline of function in healthier older persons.^21^^,^^22^ Briefly, 3075 men and women, aged 70-79 were recruited in 1997-1998 from the University of Pittsburgh and University of Tennessee Health Science Center. DXA data was obtained on participants with approval of each site’s Institutional Review Board. The cohort is multi-racial, with 45% of the women and 33% of the men identifying as Black. The follow-up period was 10 yr.

The MrOS is a multi-site, prospective cohort study designed to examine how fracture risk is related to factors, such as skeletal characteristics, lifestyle, anthropometry, physical performance, and fall propensity. MrOS enrolled 5994 men between 2000 and 2002 at 6 geographically and ethnically diverse centers in the United States (Birmingham, AL; Minneapolis, MN; Palo Alto, CA; Pittsburgh, PA; Portland, OR; San Diego, CA) with approval of each site’s Institutional Review Board. The MrOS cohort includes White (89%), Black (4%), Asian (3%), and other (3%) men.^23^ Details of the study have been described previously.^23^ Briefly, eligibility criterion included *>*65 yr of age, community dwelling, unassisted walking ability, and no bilateral hip replacements.^24^ The MrOS database includes longitudinal health information over 25 yr of follow-up, but this analysis is limited to 14 yr of follow-up associated with repeat DXA measures.

### Hip DXA

All studies captured baseline FN area, BMC, and aBMD using the manufacturer’s software and allowed the assessment of inter-individual change trajectories in those measures over 10-15 yr of follow-up. Trained technicians were employed in all studies to acquire DXA scans in accordance with clinical standards and the manufacturer of the densitometer system. The FN region was positioned consistent with the manufacturer’s protocols. Follow-up visits were conducted so the ROI size and location were consistent with the prior visit. Changes in densitometer model were associated with rigorous calibration studies and data adjustments to ensure data harmonization across the observation timeframe for each study. Data harmonization occurred within but not across studies.

For SWAN, study visits were conducted approximately annually and included hip DXA scans at each visit using Hologic instruments (Hologic, Inc.).^25^ Cross-calibration studies were conducted between sites and with system upgrades.^26^ For MBHMS**,** hip DXA scans were obtained at 12 time points (1992/93, 1993/94, 1994/95, 1995/96, 1998/99, 2000/01, 2002/03, 2005/06, 2006/07, 2007/08, 2008/09, 2009). Hip DXA scans were obtained using a GE Lunar DPX-L densitometer (GE Healthcare Technologies, Inc.) from 1992 to 2005 and GE Lunar Prodigy (GE Healthcare Technologies, Inc.) from 2005 to present. A rigorous calibration study was conducted in MBHMS at the time of densitometer upgrades to compare the machines and platforms. For Health ABC, hip DXA scans were conducted using Hologic QDR-4500 bone densitometers (Hologic Inc.) in years 1 (1997-1998), 3 (1999-2000), 5/6 (2001-2002; 2003-2004), 8 (2004-2005), and 10 (2006-2007). DXA scan reproducibility and cross-calibration across the centers was conducted following established protocols.^27^ For MrOS, hip DXA scans were performed using Hologic 4500W bone densitometers (Hologic Inc.) at 4 visits (2000-2002, 2005-2006, 2007-2009, and 2014-2016). DXA scan reproducibility was assured using uniform procedures that included centralized standardization and quality monitoring and DXA operator certification, as previously described.^28^

Because FN area, BMC and aBMD values depend on the width of the FN ROI, a data reliability study was conducted for SWAN to spot-check ROI width consistency and the extent to which consistent imaging protocols were followed (see Supplemental Methods). This reliability study confirmed a high level of protocol adherence. Although reliability studies could not be conducted for all longitudinal cohorts, strict protocol adherence policies were in effect for each cohort study, including ensuring follow-up ROI size and anatomical location, were consistent between study visits for each participant. Thus, although the number of FN ROI width adjustments cannot be known, they are suspected to be few for each longitudinal study.

### Fracture incidence

Analyses relating aBMD, area and BMC to fracture risk were limited to MrOS and Health ABC, because these older cohorts had a sufficiently large number of hip fractures to make this analysis feasible. In MrOS, participants were queried every 4 mo about fractures; when a participant reported a fracture, radiology reports were obtained (or radiographs when the fracture was uncertain) and these were centrally reviewed by a physician. In both MrOS and Health ABC, medical record adjudication was conducted for any reported fracture.^29^^,^^30^ Although fractures were also ascertained in SWAN and MBHMS, minimal trauma hip fractures were rare in these younger cohorts. Therefore, they were not included in the models of hip fracture outcomes.

### Analytic sample

**Figure 2:**
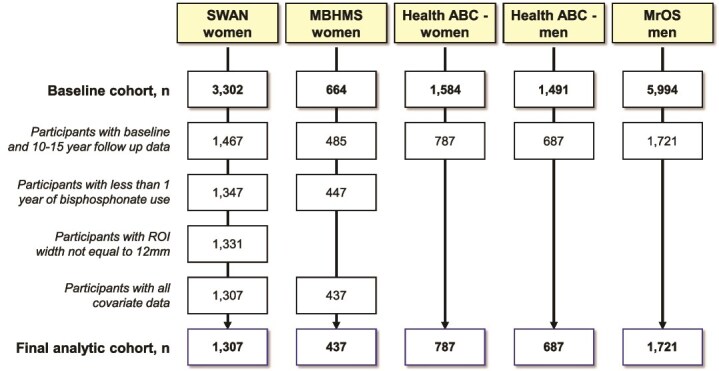
Flow chart showing the number of participants for each cohort at baseline and after exclusions for missing follow-up data, >1 yr of bisphosphonate use, ROI width < 12 mm, and missing covariate data.

Analyses were stratified by sex and cohort. The analytic samples used to calculate the annual changes in DXA parameters represented a subset of participants enrolled at baseline (Figure 2). For this analysis, participants in all cohorts were excluded, if they lacked DXA information at baseline or the 10-15 yr follow-up visit. No other exclusions were applied to the male cohorts, but additional exclusions were applied to the female cohorts. For women, exclusions included one or more years of bisphosphonate use (SWAN, *n* = 120; MBHMS, *n* = 38), FN ROI width less than 12 mm (SWAN, *n* = 16), and missing covariates (SWAN, *n* = 24; MBHMS, *n* = 10). Health ABC included information on medication use but not duration, so the >1 yr bisphosphonate-use exclusion could not be applied to Health ABC-women. SWAN was the only study to flag participants whose FN ROI width was less than 12 mm. Hormone therapy (HT) use was either adjusted in multivariate models or used as an exclusion criterion in a sensitivity analysis. The final analytic sample included 1307 women from SWAN, 437 women from MBHMS, 787 women from Health ABC, 687 men from Health ABC, and 1721 men from MrOS.

Hip DXA and demographic data obtained during the first study visit were used as baseline information for all cohorts except MBHMS. Because MBHMS enrolled women at younger ages compared to SWAN, we used baseline DXA data for MBHMS obtained within the first 4 visits and DXA data obtained 14-yr later. This flexibility in setting the baseline information within the first 4 visits allowed the average age at baseline for MBHMS women to be closer to that of SWAN women. All but 12 (1.0%) SWAN and 114 (26.4%) MBHMS women were post-menopausal by the 14-15 yr follow-up visit.

### Statistical analysis

Descriptive statistics of demographic and body size characteristics as well as baseline and group average annual changes in FN area, BMC, and aBMD were examined for each cohort. Annualized changes in each FN DXA parameter were calculated for each individual as the total change in FN area, BMC, and aBMD between the first and follow-up visit and divided by the duration of the observation period. The individual annual changes in DXA parameters were averaged for each cohort. The demographic, baseline DXA parameters, and annual changes in DXA parameters were compared among cohorts using a one-way ANOVA with Tukey post-hoc test for the 3 female cohorts and a *t*-test for the 2 male cohorts.

The first analysis tested for associations between baseline FN area and total change in DXA parameters using the final analytical cohort (Figure 2). Correlations were obtained between total changes in FN area, BMC, and aBMD and height-adjusted baseline FN area within each cohort. Height-adjusted baseline FN area was used to take body size effects into consideration because FN area correlates with height.^11^ Height-adjusted baseline FN area was obtained for women and men separately for each cohort from the residuals of sex-specific linear regressions of baseline FN area on baseline height. Next, separate linear regression models were run for each cohort to examine the association of height adjusted baseline FN area with the outcomes of total change in FN area, BMC, and aBMD (each a separate model) adjusted for race/ethnicity, HT use at baseline (SWAN, MBHMS) or more than 1 yr (Health ABC-women), total weight change, and baseline age, weight, height, and aBMD. Adjusted *R*^2^ values were obtained from each model.

To investigate the mechanisms by which baseline external FN size might be associated with annualized changes in FN area, BMC, and aBMD, women and men were grouped into cohort-stratified tertiles based on their height-adjusted baseline FN area (narrow, intermediate, and wide). To be consistent with our prior work, baseline FN area was adjusted for height to take body stature into consideration since outer bone size tends to increase with height.^11^ Determination of tertiles was conducted for each cohort separately. For SWAN and MHBMS, women were sorted into tertiles using the final analytic cohorts (ie, after exclusions) resulting in similar numbers of individuals in each tertile. For Health ABC and MrOS, sorting occurred using the entire cohort followed by exclusion resulting in minor differences in the number of individuals in each tertile. Baseline characteristics (age, height, and weight), baseline and annual change in DXA parameters, proportion of individuals in the self-identified race/ethnic groups, HT use (women) were compared among height adjusted baseline FN area tertiles using ANOVA. Chi-square goodness-of-fit test or Fisher’s exact test were used to assess statistically significant differences between height-adjusted baseline area tertiles for race/ethnicity, and hormone use (women). The annualized changes in FN area, BMC, and aBMD were adjusted for race/ethnicity and baseline age and compared using ANCOVA (SWAN, MBHMS) or general linear model (MrOS, Health ABC) to determine if there were statistically significant differences between the tertiles.

### Fracture risk

Cox proportional-hazard models were used to test whether height adjusted baseline FN area was associated with hip fracture risk. Hazard ratios (HR) were calculated for Health ABC-women, Health ABC-men, and MrOS using the baseline cohort (Figure 2). The Health ABC-women, Health ABC-men, and MrOS cohorts were separated into height-adjusted baseline FN area tertiles. Hazard ratios for fracture were calculated for the intermediate and widest tertiles with the narrowest tertile serving as the referent group and tested if hip fracture risk differed among the tertiles. Models were conducted with and without adjustments for baseline BMC. For MrOS, White vs non-White was used to adjust HR for race/ethnicity because of the small number of Non-White men. Finally, each height-adjusted baseline area tertile in MrOS was further divided into FN BMC tertiles (low, intermediate, and high) to create 9 subgroups. Hazard ratios were calculated and compared across the 9 subgroups to investigate how differences in hip fracture risk depended on different combinations of baseline FN area and BMC. The narrow area+high BMC group served as the reference group for this analysis. This secondary analysis was not conducted for Health ABC-women and Health ABC-men cohorts, because there were no hip fractures in the reference group (narrow area + high BMC group).

## Results

### Cohort characteristics

Baseline characteristics and the annual changes in FN area, BMC, and aBMD are shown in Table 1 for participants included in each of the final analytic cohorts. The SWAN and Health ABC cohorts were racially and ethnically diverse, in contrast to the MBHMS and MrOS cohorts which were comprised primarily (>90%) of non-Hispanic White women and men, respectively. The average baseline age confirmed representation of midlife women in SWAN and MBHMS and older women and men in Health ABC and MrOS (Figure 1). Group averages for the annual change in FN aBMD were approximately 2 times greater for midlife women compared to older women and men, as expected (Table 1).

**Table 1:** Baseline characteristics, annual changes in FN DXA measures, and ethnicity composition for the 4 analytic cohorts (for those have data at baseline and last study visit).

	**SWAN women (*n* = 1307)**	**MBHMS women (*n* = 437)**	**Health ABC-women (*n* = 787)**	**Health ABC-men (*n* = 687)**	**MrOS men (*n* = 1721)**
**Characteristic**					
** Baseline age (yr)**	**46.3 (2.6)** ^**b,c**^	**40.1 (5.0)** ^**a,c**^	**73.1 (2.8)** ^**a,b**^	73.4 (2.8)^*^	70.4 (4.2)^*^
** Baseline weight (kg)**	**72.7 (18.2)** ^**c**^	**73.8 (17.5)** ^**c**^	**69.9 (13.8)** ^**a,b**^	82.0 (13.0)^*^	84.0 (12.3)^*^
** Baseline height (cm)**	**162.7 (6.5)** ^**c**^	**163.4 (6.0)** ^**c**^	**159.7 (6.2)** ^**a,b**^	173.6 (6.7)^*^	175.3 (6.7)^*^
** Baseline BMI (kg/m^2^)**	27.4 (6.5)	27.6 (6.3)	27.4 (5.1)	27.2 (3.8)	27.3 (3.5)
** Baseline aBMD (g/cm^2^)**	**0.85 (0.13)** ^**b,c**^	**1.00 (0.14)** ^**a,c**^	**0.69 (0.12)** ^**a,b**^	0.80 (0.13)	0.80 (0.12)
** Baseline BMC (g)**	**4.04 (0.68)** ^**b,c**^	**4.75 (0.81)** ^**a,c**^	**3.37 (0.67)** ^**a,b**^	4.55 (0.82)	4.52 (0.73)
** Baseline area (cm^2^)**	**4.78 (0.38)** ^**b,c**^	**4.57 (0.34)** ^**a,c**^	**4.88 (0.51)** ^**a,b**^	5.71 (0.52)	5.68 (0.42)
** Annual change aBMD (g/cm^2^/yr)**	**−0.006 (0.005)** ^**c**^	**−0.006 (0.006)** ^**c**^	**−0.003 (0.006)** ^**a,b**^	−0.004 (0.007)^*^	−0.003 (0.005)^*^
** Annual change BMC (g/yr)**	**−0.023 (0.025)** ^**b,c**^	**−0.018 (0.036)** ^**a**^	**−0.014 (0.039)** ^**a**^	−0.026 (0.050)^*^	−0.016 (0.031)^*^
** Annual change area (cm^2^/yr)**	**0.007 (0.015)** ^**b,c**^	**0.024 (0.020)** ^**a,c**^	**0.001 (0.028)** ^**a,b**^	−0.002 (0.030)^*^	0.004 (0.017)^*^
**Ethnicity**					
** White (non-Hispanic) (%)**	643 (49.2%)	437 (100%)	487 (61.9%)	486 (70.7%)	
** White (Hispanic+non-Hispanic) (%)**					1561 (90.7%)
** Black (%)**	360 (27.5%)		300 (38.1%)	201 (29.3%)	52 (3.0%)
** Chinese (%)**	156 (11.9%)				
** Japanese (%)**	148 (11.3%)				
** Asian (%)**					55 (3.2%)
** Native Hawaiian (%)**					2 (0.1%)
** American Indian (%)**					2 (0.1%)
** Multi-racial (%)**					21 (1.2%)
** Unknown (other) (%)**					28 (1.6%)

Data are shown as mean (SD). Ethnicity data are shown as number of individuals (percent of overall cohort).

Bold font. For males,

^*^ = *p* < .05 (*t*-test);

^#^for females, *p* < .05 (ANOVA),

^a^ = different from SWAN,

^b^ = different from MBHMS,

^c^ = different from Health ABC.

### Association between baseline FN area and changes in DXA parameters

Height-adjusted baseline FN area was negatively correlated with the changes in FN area and BMC, but not aBMD, over 10-15 yr for women and men (Table 2). Correlations were consistent across the 4 analytic cohorts, except for MBHMS, which showed a weak but statistically significant association between baseline area and the 14-yr change in aBMD. Baseline area remained a statistically significant predictor of the 10-15 yr changes in FN area and BMC for SWAN, MBHMS, Health ABC, and MrOS after adjusting for race/ethnicity, baseline hormone treatment use (SWAN, MBHMS, Health ABC-women), weight change, and baseline age, weight, height, and aBMD (Table S1). These associations did not change after excluding women with baseline HT-use (*n* = 2, SWAN women only; data not shown). The negative correlations shown in Table 2 indicated that individuals with larger baseline FN area showed smaller increases in FN area over 10-15 yr. The negative correlations for FN BMC indicated that individuals with larger baseline FN area showed greater losses in BMC over 10-15 yr.

**Table 2:** Correlations between total change in FN area, BMC, and aBMD with baseline FN area-height residuals for (A) SWAN women, (B) MBHMS women, (C) Health ABC-women, (D) Health ABC-men, and (E) MrOS men.

**DXA measure**	**Correlation (95% CI)**	***R*** ^**2**^	***p*-value**
**A. SWAN women**			
**Area**	**−0.37 (−0.41, −0.32)**	**0.135**	**<.001**
**BMC**	**−0.23 (−0.28, −0.18)**	**0.053**	**<.001**
**aBMD**	0.02 (−0.04, 0.07)	0.0003	.529
**B. MBHMS women**			
**Area**	**−0.36 (−0.44, −0.28)**	**0.132**	**<.001**
**BMC**	**−0.26 (−0.35, −0.17)**	**0.069**	**<.001**
**aBMD**	**−0.19 (−0.28, −0.10)**	**0.037**	**<.001**
**C. Health ABC Women**			
**Area**	**−0.28 (−0.35, −0.22)**	**0.080**	**<.001**
**BMC**	**−0.12 (−0.19, −0.06)**	**0.015**	**<.001**
**aBMD**	0.07 (−0.004, 0.14)	0.004	.064
**D. Health ABC Men**			
**Area**	**−0.27 (−0.34, −0.20)**	**0.071**	**<.001**
**BMC**	**−0.19 (−0.27, −0.12)**	**0.038**	**<.001**
**aBMD**	−0.03 (−0.10, 0.05)	0.0007	.495
**E. MrOS men**			
**Area**	**−0.31 (−0.35, −0.27)**	**0.096**	**<.001**
**BMC**	**−0.14 (−0.19, −0.09)**	**0.020**	**<.001**
**aBMD**	0.02 (−0.03, 0.07)	0.0004	.413

Significant correlations are shown in bold font.

Abbreviations: Health ABC, Health, Aging, and Body Composition Study; MBHMS, Michigan Bone Health and Metabolism Study; MrOS, Osteoporotic Fractures in Men Study; SWAN, Study of Women’s Health Across the Nation.

### Height-adjusted FN area tertiles

A comparison of the baseline characteristics of women and men sorted into height-adjusted baseline FN area tertiles is shown in Table S2. For all cohorts, baseline FN BMC varied among the tertiles with BMC increasing progressively from the narrowest to the widest tertiles. Despite having greater BMC, individuals in the widest tertile showed lower aBMD compared to those in the narrowest tertile for SWAN, Health ABC, and MrOS but not MHBMS.

Sorting the analytical cohorts using baseline FN aBMD resulted in tertiles with qualitatively different sets of characteristics (Table S3) compared to sorting using baseline FN area. Women and men in the low aBMD tertile were shorter, less heavy, and had a combination of lower BMC and higher area compared to those in the higher aBMD tertile which had a combination of higher BMC and lower area. In contrast, sorting using baseline FN area resulted in individuals in the narrowest tertile showing a combination of lower BMC and lower area compared to those sorted into the widest tertile who showed higher BMC and higher area. This qualitative comparison suggested that sorting individuals using baseline FN area resulted in different subgroups compared to sorting using aBMD.

### Tertile-specific changes in DXA parameters over 10-15 yr

**Figure 3:**
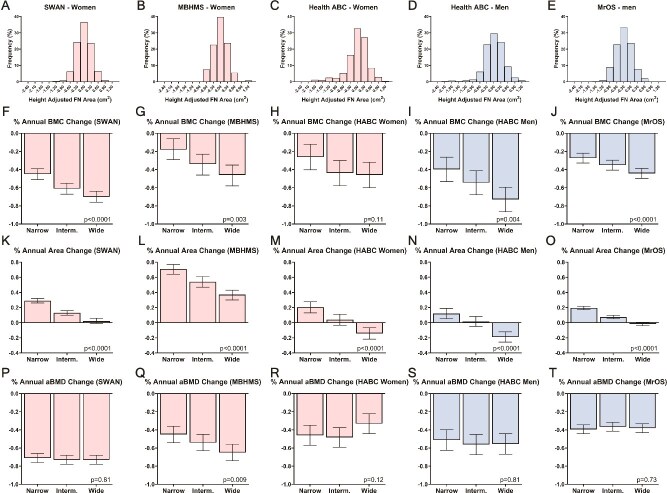
Percent annual change in hip BMC, area, and aBMD measured over 10-15 yr of follow-up differed among tertiles of height adjusted baseline area for 5 cohorts of women and men. Error bars are 95% CIs.

The age and race and ethnicity adjusted annual changes in area and BMC over the 10-15 yr of follow-up differed across height-adjusted baseline FN area tertiles for women and men (Figure 3), as hypothesized. In both cohorts of menopausal women, those in the narrowest tertile had smaller FN BMC declines but greater area increases, whereas those in the widest tertile had greater FN BMC declines and lower area increases. The tertile-specific differences observed for MBHMS and SWAN women were observed for older women in Health ABC for changes in FN area but not BMC. Older men in MrOS and Health ABC showed tertile-specific differences in FN BMC-change and area-change consistent with those observed for menopausal women. In both MrOS and Health ABC, men in the narrowest tertile had lower FN BMC declines but greater area increases, whereas men in the widest tertile had greater FN BMC declines and lower area increases. The annual FN BMC loss of the widest tertile was nearly double that of the narrowest tertile for most cohorts. As hypothesized, the annual change in FN aBMD did not differ across tertiles for SWAN, Health ABC-women, Health ABC-men, and MrOS. MBHMS was the only cohort to show a difference in annual FN aBMD change.

### Fracture risk

**Figure 4:**
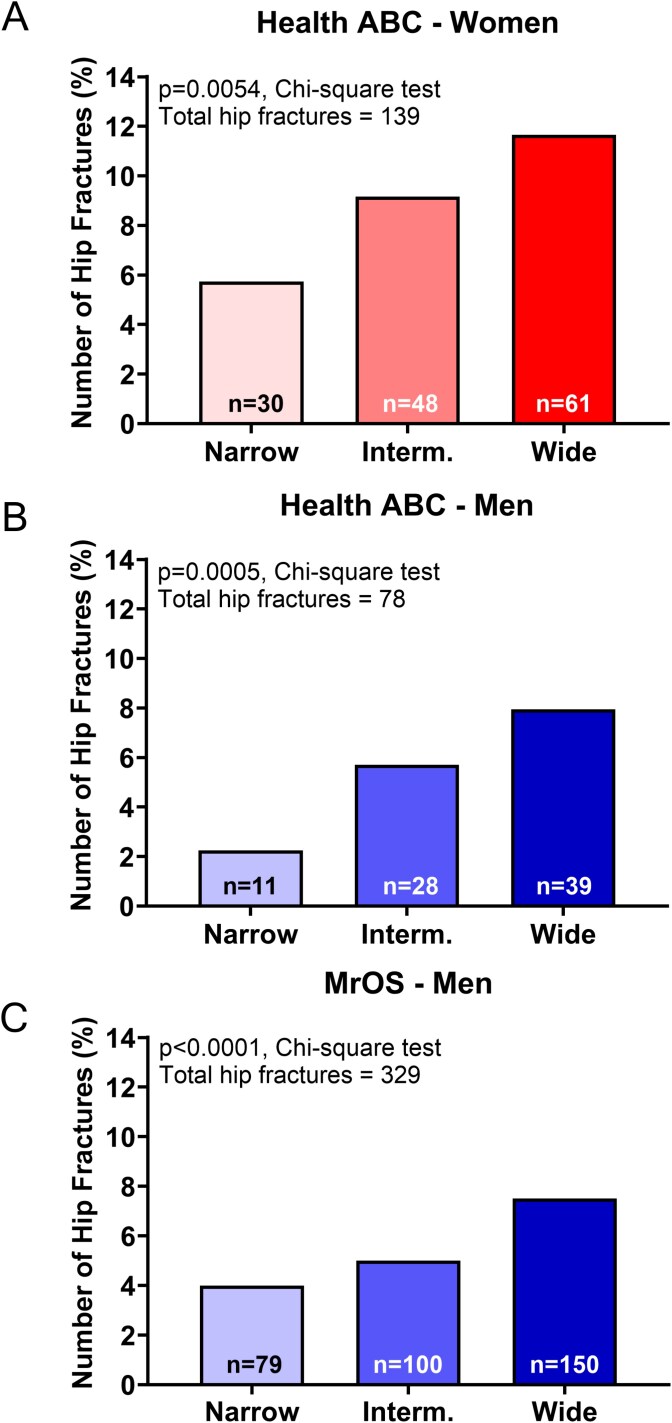
The number (%) of hip fractures increased progressively from the narrow to the wide height adjusted baseline area tertile for (A) Health ABC-women, (B) Health ABC-men, and (C) MrOS men.

Incident hip fractures numbered 139 among Health ABC-women, 78 among Health ABC-men, and 329 among the men in MrOS over the 10-14 yr of follow-up for these 3 cohorts. The number of hip fractures increased progressively from the narrowest to the widest tertiles for each of the 3 cohorts (Figure 4). The first fracture outcome analysis tested if the HR depended on baseline predictors of hip fracture risk after adjusting for age and race/ethnicity. Health ABC-women in the widest tertile were 1.70 (95% CI 1.09, 2.64) times more likely to fracture a hip compared to the narrowest tertile (Table 3A). Adjustment for BMC increased the magnitude of the association to 2.54 (95% CI 1.60, 4.02) (widest vs narrowest tertiles). Similarly, men in the widest tertile were 3.14 (95% CI 1.59, 6.21) (Health ABC-men, Table 3B) and 1.74 (95% CI 1.32, 2.29) (MrOS, Table 3C) times more likely to have a hip fracture compared to men in the narrowest tertile. Adjustment for FN BMC increased the strength of the associations to 4.21 (95% CI 2.09, 8.50) and 2.43 (95% CI 1.83, 3.21) for Health ABC-men and MrOS, respectively.

**Table 3:** Hazard ratios (95% CI) calculated for baseline bone area predicting future hip fractures for (A) Health ABC-women, (B) Health ABC-men, and (C) MrOS men.

**Height-adj. area tertile**	**Age + Race adjusted**	**Age + Race + BMC adjusted**
**A. Health ABC–Women hip fractures (*N* = 1570 participants; *n* = 139 hip fractures)**		
** Narrow**	1.00 (reference)	1.00 (reference)
** Intermediate**	1.31 (0.83, 2.08)	**1.76 (1.10, 2.83)**
** Wide**	**1.70 (1.09, 2.64)**	**2.54 (1.60, 4.02)**
**B. Health ABC–Men hip fractures (*N* = 1472 participants; *n* = 78 hip fractures)**		
** Narrow**	1.00 (reference)	1.00 (reference)
** Intermediate**	**2.29 (1.13, 4.62)**	**2.71 (1.32, 5.55)**
** Wide**	**3.14 (1.59, 6.21)**	**4.21 (2.09, 8.50)**
**C. MrOS hip fractures (*N* = 5594 participants; *n* = 329 hip fractures)**		
** Narrow**	1.00 (reference)	1.00 (reference)
** Intermediate**	1.21 (0.90, 1.63)	**1.43 (1.07, 1.93)**
** Wide**	**1.74 (1.32, 2.29)**	**2.43 (1.83, 3.21)**

Bold numbers indicate significant Hazard Ratios. Abbreviations: Health ABC, Health, Aging, and Body Composition Study; MrOS, Osteoporotic Fractures in Men Study.

For the second fracture risk analysis, each height-adjusted baseline area tertile for MrOS was further sorted into low, intermediate, and high baseline FN BMC tertiles creating 9 subgroups. Hazard ratios were compared across the 9 subgroups using the low area+high BMC subgroup as the reference (Table 4). Having low baseline BMC was a risk factor for all FN area tertiles, as expected. Importantly, at any given FN BMC, the risk of fracture progressively increased from narrow to intermediate to wide tertiles. Critically, men in the widest tertile were significantly more likely to have a hip fracture for all BMC tertiles when compared to the low area+high BMC subgroup (referent group). Hazard ratios for the wide tertile ranged from 4.1 (95% CI 1.5-11.8) for those with high FN BMC to 17.1 (95% CI 6.2-46.8) for those with low FN BMC. Notably, significant HR values were identified for subgroups showing T-scores in the normal (T-score ≥ −1.5) and osteopenic (−2.5 < T-score < −1.5) ranges.

**Table 4:** Hazard ratios (HR) and upper and lower 95% CIs for MrOS hip fractures sorted into height-adjusted area tertiles and then into BMC tertiles, adjusted by age and race (non-White vs White).

**Tertile**		**Low BMC**	**Intermediate BMC**	**High BMC**
**Narrow**	**HR**	**8.7 (3.2–24.0)**	2.8 (0.9–8.4)	1 (referent)
	**aBMD** **T-score**	0.70 (0.07) −1.31 (0.59)	0.82 (0.05) −0.28 (0.43)	0.97 (0.09) 0.94 (0.76)
**Intermediate**	**HR**	**10.2 (3.7–28.1)**	**4.7 (1.7–13.5)**	2.6 (0.9-8.0)
	**aBMD** **T-score**	0.66 (0.06) −1.67 (0.48)	0.77 (0.04) −0.70 (0.34)	0.92 (0.09) 0.49 (0.75)
**Wide**	**HR**	**17.1 (6.2–46.8)**	**7.6 (2.7-21.2)**	**4.1 (1.5-11.8)**
	**aBMD** **T-score**	0.62 (0.06) −2.00 (0.48)	0.73 (0.04) −1.09 (0.35)	0.87 (0.09) 0.10 (0.78)

Significant differences in HR values from the referent group are shown in bold font. The average aBMD and T-score and SDs for each of the 9 subgroups are also shown.

Abbreviations: Health ABC, Health, Aging, and Body Composition Study; MrOS, Osteoporotic Fractures in Men Study.

## Discussion

Analysis of hip DXA images collected over 10-15 yr support the hypothesis that baseline FN area measured from routine hip DXA scans is associated with different annual losses of FN BMC and annual increases in FN area that are not consistently reflected in annual losses of FN aBMD. These results replicated our prior work^11^ in larger cohorts of midlife women and now establish that similar heterogenous structural changes also occur in older women and men. Femoral neck area measured from DXA correlated with FN width,^31^ which suggests the different trajectories of BMC loss and area gain are associated with baseline external bone size. Although hip strength was not assessed, the 2-fold greater hip fracture incidence of older women and men in the widest tertile compared to the narrowest tertile suggested that the different structural changes in the 2 groups were associated with different trajectories of fracture resistance decline.^15^^,^^16^ Critically, the different structural changes and fracture incidence among the FN area tertiles were not consistently reflected in FN aBMD loss. Sorting individuals using baseline FN area resulted in tertiles with different combinations of BMC and area values compared to sorting using baseline aBMD. Thus, our outcomes provided a novel view of age-related structural changes occurring within the FN. These data showed for the first time that unique patterns of age-related structural changes with different biomechanical implications exist within populations of women and men, are associated with baseline external bone size, and are not reflected in changes in FN aBMD.

A major outcome of this study was the demonstration that different trajectories of BMC loss and area gain were inconsistently reflected in changes in FN aBMD. The patterns were observed in 5 longitudinal cohorts over 10-15 yr of follow-up, providing evidence that distinct subgroups undergo unique structural changes affecting the proportion of bone loss to area gain. With aBMD calculated as a ratio of BMC to area, the different proportions of bone loss to area gain among the subgroups resulted in similar aBMD losses. These results suggest that assessing baseline FN area, which is available on DXA outputs, may provide useful information about subsequent changes in strength and may help inform clinicians about underlying structural changes contributing to losses in aBMD for their patients.

Prior studies have generally sought to identify a single trait or group of traits^17^ that could predict fracture risk for an entire population. Our data showing different trajectories of structural change (Figure 3) and different fracture incidence (Figure 4), indicate there are multiple structural pathways to increased fracture risk within a population, each represented by a unique set of traits.^15^^,^^16^ The modest but significant *R*^2^ values shown in Table 2 may reflect the low power that arises when attempting to identify a consistent structural measure for a heterogeneous population. Subgroup analyses, which involve identifying individuals that tend to be more similar within a group but different from other groups, have proven clinically useful for refining disease diagnoses in other fields^32^ supporting the concept of population heterogeneity and that group-level averages do not represent individual-level responses.^33^ Although the subgroup analysis used herein provided a rigorous scientific basis to reveal the underlying heterogeneity, we recognize nested subgroup stratification may not be practical clinically and that alternative, multi-factorial approaches^34^ may be needed to identify individuals with lower than expected bone strength within a heterogeneous population.

Different combinations of bone structural traits representing different fracture-risk pathways have been identified previously using unsupervised sorting methods.^35–39^ In contrast to these studies, we identified subgroups using a priori knowledge that external bone size is associated with different combinations of traits.^11^ Because bone is a complex adaptive system, multiple traits are coordinately adjusted during growth, so the resulting combination of traits defines whether the adult bone is strong and fracture resistant. This coordination of traits, observed primarily for diaphyseal bone, translates to cortico-cancellous structures like the FN.^11^ Narrower bones adjust mass, porosity, and material properties during growth to maximize adult bone strength, otherwise the small width would make this phenotype weak. With aging, the narrowest tertiles showed lower FN BMC loss and greater area gains (Figure 3). This pattern of structural change suggests strength is being maintained or possibly improved with aging for those in the narrowest tertile despite the loss of FN aBMD. Thus, the concept that narrow bones coordinately adjust traits to maximize strength during growth carries over into aging.

In contrast, wider bones have a larger external size to provide strength but minimize mass (BMC) accumulation during growth to avoid being heavy.^35^ With aging, individuals in the wide tertile lost FN BMC with minimal area gain suggesting wider FN may experience structural changes over 10-15 yr leading to more pronounced losses in strength than bones with narrower FN.^15^ This loss in strength was sufficiently large such that women and men in the widest tertile were 2-3 times more likely to fracture a hip. Thus, a priori sorting of individuals using external size may offer the benefit of predicting different annual changes in structural parameters, whereby narrower FNs maximize strength with aging, whereas wider FNs minimize mass. The available hip DXA data do not provide structural details, so we do not yet know the specific structural changes responsible for the fracture incidence within each FN area tertile nor how they differ. We would expect well-defined age-related structural changes to occur in each subgroup, such as cortical thinning, increased porosity, reduced trabecular mass, and increased tissue-brittleness.^40^ However, we expect the timing and magnitude of these structural changes will differ among the subgroups.^16^ The only cohort to not show a similar trend for BMC-loss was Health ABC women. Given the lack of information on osteoporosis drug treatment for this cohort, we cannot rule out the possibility that the slightly blunted BMC loss of the wider tertile may reflect treatment effects with anti-resorptive medications in this subgroup.

Prior studies examining the association between external bone size and fracture risk have reported inconsistent outcomes, such that increased fracture risk has been reported for individuals with narrow bones,^41^^,^^42^ wide bones,^43^ or no difference in external size.^44^ These prior studies generally identified structural measures contributing to fracture risk by comparing cases to controls or higher-risk to lower-risk groups, assuming a single trait could predict fracture risk for an entire population. We approached this problem differently by testing the hypothesis that each FN area tertile would show different structural changes over time. Our findings, which support the hypothesis, provide evidence that the relative amounts of bone loss and area gain vary significantly among individuals. Given the heterogeneous structural changes reported herein, a “one size fits all” approach may not adequately reflect strength decline for populations of women and men. These findings offer novel insights and provide the opportunity to improve diagnostic measures based on a different set of traits to predict fracture risk for a population.

Our data showed that greater BMC declines did not correspond to greater area increases,^45^ which suggests the general assumption that bone loss elicits compensatory periosteal expansion needs further investigation.^13^^,^^46^ Given the different relative proportions of FN BMC loss to area gain among the tertiles, we would expect loss of aBMD may reflect loss of strength for the widest tertile but not for the narrowest tertile. These outcomes may help explain why some studies showed repeat aBMD measures provide no additional insight into fracture risk beyond that provided by baseline aBMD when examined at the population level.^4^ Examining BMC and area separately provided a novel view of how different combinations of BMC and area contributed to fracture risk in MrOS men. Men with wide FN combined with low or intermediate BMC were ~9-17 times more likely to fracture a hip. Individuals with a combination of wide FN and low BMC had a low aBMD and T-score, which reflected the increased fracture risk for this subgroup, as expected. However, there were subgroups with combinations of BMC and area values for which aBMD and T-scores may be less discerning regarding fracture risk. For example, the subgroup with wide FN + high BMC had the third largest aBMD and T-score values but also showed a 4.1-fold higher fracture risk. Likewise, the subgroup with narrow FN + intermediate BMC had a slightly lower aBMD and T-score as the wide + high BMC subgroup but the HR was not significant. This data suggested that low aBMD and T-scores may reflect increased fracture risk for some individuals, but that increased fracture risk can arise for individuals with aBMD and T-scores in the normal range. Thus, examining HR values relative to the combination of FN area and FN BMC values, rather than aBMD alone, showed how aBMD and T-scores are inconsistently associated with fracture risk across a population.

Annual changes in bone structure were quantified from FN DXA aBMD, BMC, and area measures available at baseline and 10-15 yr of follow-up. We focused on the total change in hip DXA parameters over 10-15 yr of follow-up, recognizing that further refinement of these outcomes is possible since changes in these parameters may not be linear with aging.^47^^,^^48^ Further, the underlying cellular and histological bases contributing to the different structural trajectories is currently unknown but are part of ongoing investigations. The differences in BMC loss and area gain among the tertiles was prominent in mid-life women and present in older men but showed weakening of the pattern in older women. Femoral neck area is a measure of external bone size^31^ and increases in FN area suggest an expansion of the periosteal surface. Finding negative annual changes in FN area for women and men in the widest tertile of Health ABC was surprising. Based on our experience^31^ and given the greater loss of BMC in the widest tertile, we suspect these negative area changes reflect loss of X-ray attenuation in the superior FN region, which typically shows large trabecular bone loss, and the ability of edge detection algorithms to identify the superior surface consistently over time. This effective loss of FN width arising from excessive osteoclastic resorption of the cortex^49^ and/or trabecular mass of the tensile arcade^50^ for those in the widest tertile may accelerate the strength decline for these individuals. Finally, we cannot rule out errors due to accuracy or reproducibility, but these would be expected to be random within tertiles and not to explain the tertile-specific annual BMC and area changes shown in Figure 3.

The implications of these unique patterns of structural changes for clinical management of fracture risk must be established and may be important. The association between baseline external bone size and subsequent age-related structural and strength changes could provide the opportunity to identify individuals at eventual increased fracture risk at an earlier stage. Monitoring strategies may be possible to discern if aBMD loss is due to losses in BMC vs loss in aBMD arising primarily from large increases in area. Identifying those differences could conceivably, in turn, help inform clinicians on treatment strategies as aBMD loss due to losses in BMC may be ideally treated differently compared to losses in aBMD arising primarily from large increases in area. Further, targeted interventions may be possible to reduce fracture risk. For example, given the significantly greater fracture risk of those in the widest tertile, one could argue the value of establishing new intervention programs aimed at slowing the large FN BMC decline for those in the widest tertile to help maintain strength and to reduce fracture risk. Whether the different trajectories of BMC loss and area gains among the FN area tertiles are associated with different responses to available treatment approaches has yet to be determined. An understanding of the cellular underpinnings of different size trajectories would provide an understanding of how the choice of therapies could optimize bone strength.

Implementation of external bone size measures in fracture assessment and intervention decision making would not require the development of new technologies since FN area is available in DXA images. Finding that baseline FN area was associated with different trajectories of change in FN area and BMC does not imply that monitoring strategies should focus exclusively on periosteal expansion, which would be challenging given that small dimensional changes in external size may be difficult to detect using clinical imaging systems. Our results suggest additional research is warranted to operationalize how baseline bone size could be used to sort populations into subgroups and then monitor individuals using routine FN DXA BMC for greater than expected bone loss, which we show varies among the subgroups with greater annual losses for those in the wide subgroup. Improvements in assessing external bone size from DXA and establishing external size distribution curves for populations would be appropriate next steps for the implementation of these approaches.

In conclusion, baseline FN area measured from routine hip DXA scans was associated with different annual losses of FN BMC and annual increases in FN area that were not consistently reflected in annual losses of FN aBMD. The different structural changes may help to explain why individuals with wider FNs showed significantly greater fracture risk compared to individuals with narrower FNs. These results provide evidence that age-related structural changes are heterogeneous within populations of women and men. Based on prior studies assessing long bone strength^11^^,^^12^ and the greater fracture risk of the wide compared to the narrow tertile reported herein, our data suggest that any strength advantage of wider FNs at baseline may be lost with aging. In contrast, our data also suggest that the strength of narrower FNs may be maintained with aging despite showing a loss of aBMD. The results of this study strongly advocate for developing new diagnostic measures that reflect these unique structural changes to more consistently monitor changes in bone health with aging. Whether these unique patterns of structural change are associated with different responses to interventions has yet to be determined.

## Supplementary Material

Bone_Width_Hip_FX_JBMR_Jepsen_Supplementary_Data_REVISED_clean_zjaf090

## Data Availability

Deidentified data are available upon request for each of the longitudinal cohorts.
