# Supplementary material for: Femoral neck width is associated with unique trajectories of age-related hip structural changes and fracture risk within populations of adult women and men
Source: J Bone Miner Res. 2025 Jun 28;40(10):1114–26. doi: 10.1093/jbmr/zjaf090 (PMC12487784; doi:10.1093/jbmr/zjaf090)
Supplement: Bone_Width_Hip_FX_JBMR_Jepsen_Supplementary_Data_REVISED_clean_zjaf090 [file bone_width_hip_fx_jbmr_jepsen_supplementary_data_revised_clean_zjaf090.docx]

**Supplemental Method**

**Method**

*Data reliability:* A small data reliability study was conducted for the SWAN study to spot-check ROI size consistency and to understand the extent to which consistent imaging protocols were followed. The default width of the FN ROI was 15 mm, but this width could be adjusted to accommodate positioning errors or interference of the ischium with the FN ROI. For SWAN, the FN ROI width was not recorded in the database except when it was adjusted to 12 mm or less. SWAN women for whom the ROI width was adjusted to <12 mm were removed from the analysis (n=11) as this adjustment lead to unacceptably large reductions in all measures. Because unrecorded ROI width adjustments could lead to DXA parameter values that reflect ROI width rather than variation associated with external-size or age, the data reliability study conducted for SWAN assessed adherence to using the default FN ROI. First, FN ROI widths were manually recorded for the baseline scans of 377 randomly selected women from the Michigan (n=177) and Pittsburgh (n=200) sites. It was found that 376 of the 377 scans had a baseline ROI width of 15 mm; 1 woman had an ROI width of 12 mm, which was removed from the study. Second, FN ROI widths were manually recorded for study visits 0-5 and 10-16 (files for visits 7-9 were not available) for 40 randomly selected women from the Michigan site. The ROI width was 15 mm for 449 of 452 visits (99.3%); ROI width was adjusted to 14 mm for three individuals at only one of their visits. This data reliability study confirmed a high level of protocol adherence for SWAN. Although a data reliability study could not be conducted for MrOS and Health ABC, similar strict protocol adherence policies were in effect. Thus, although the number of FN ROI width adjustments cannot be known, they are suspected to be few for each longitudinal study.

**Supplemental Tables**

**Table S1.** Multivariable regression for predicting changes in bone area, BMC, and aBMD over 10-15 years of follow up for A) SWAN women, B) MBHMS women, and C) Health ABC women, D) Health ABC men, and E) MrOS men. Significant predictors are shown in bold font.

**A1.** Predictors of 15-year change in area for SWAN women

| **Multivariate Model** | **Estimate (95% CI)** | **Standardized β** | **p-value** |
| --- | --- | --- | --- |
| Race/ethnicity |  |  |  |
| **Black** | **-0.037 (-0.068, -0.007)** | **-0.071** | **0.017** |
| Chinese | -0.022 (-0.064, 0.020) | -0.030 | 0.305 |
| Japanese | 0.004 (-0.038, 0.047) | 0.006 | 0.846 |
| White | ref | ref | ref |
| HT-use | 0.147 (-0.157, 0.450) | 0.025 | 0.344 |
| Baseline age | 0.000 (-0.005, 0.004) | -0.006 | 0.831 |
| Baseline weight | 0.000 (-0.001, 0.001) | 0.024 | 0.501 |
| **Baseline height** | **0.008 (0.006, 0.011)** | **0.233** | **<0.001** |
| Baseline aBMD | 0.013 (-0.111, 0.136) | 0.007 | 0.840 |
| **Baseline area** | **-0.272 (-0.310, -0.234)** | **-0.440** | **<0.001** |
| Change in weight | -0.001 (-0.002, 0.000) | -0.038 | 0.152 |
|  |  |  |  |
| Adjusted R^2^ | 0.135 |  |  |

**A2.** Predictors of 15-year change in BMC for SWAN women

| **Multivariate Model** | **Estimate (95% CI)** | **Standardized β** | **p-value** |
| --- | --- | --- | --- |
| Race/ethnicity |  |  |  |
| Black | 0.016 (-0.032, 0.064) | 0.018 | 0.513 |
| Chinese | 0.046 (-0.019, 0.112) | 0.039 | 0.164 |
| **Japanese** | **-0.131 (-0.198, -0.064)** | **-0.107** | **<0.001** |
| White | ref | ref | ref |
| HT-use | 0.250 (-0.226, 0.725) | 0.025 | 0.303 |
| Baseline age | -0.006 (-0.013, -0.001) | -0.043 | 0.081 |
| **Baseline weight** | **0.003 (0.001, 0.004)** | **0.121** | **<0.001** |
| **Baseline height** | **0.005 (0.002, 0.009)** | **0.089** | **0.004** |
| **Baseline aBMD** | **-0.806 (-0.999, -0.613)** | **-0.263** | **<0.001** |
| **Baseline area** | **-0.301 (-0.360, -0.241)** | **-0.293** | **<0.001** |
| **Change in weight** | **0.016 (0.014, 0.018)** | **0.355** | **<0.001** |
|  |  |  |  |
| Adjusted R^2^ | 0.232 |  |  |

**A3.** Predictors of 15-year change in aBMD for SWAN women

| **Multivariate Model** | **Estimate (95% CI)** | **Standardized β** | **p-value** |
| --- | --- | --- | --- |
| Race/ethnicity |  |  |  |
| Black | 0.008 (0.000, 0.017) | 0.053 | 0.054 |
| **Chinese** | **0.013 (0.002, 0.025)** | **0.061** | **0.024** |
| **Japanese** | **-0.027 (-0.039, -0.015)** | **-0.122** | **<0.001** |
| White | ref | ref | ref |
| HT-use | 0.015 (-0.070, 0.099) | 0.008 | 0.732 |
| Baseline age | -0.001 (-0.003, 0.000) | -0.047 | 0.051 |
| **Baseline weight** | **0.000 (0.000, 0.001)** | **0.119** | **<0.001** |
| Baseline height | 0.000 (-0.001, 0.000) | -0.015 | 0.631 |
| **Baseline aBMD** | **-0.188 (-0.223, -0.154)** | **-0.338** | **<0.001** |
| Baseline area | -0.001 (-0.012, 0.009) | -0.007 | 0.820 |
| **Change in weight** | **0.003 (0.003, 0.004)** | **0.419** | **<0.001** |
|  |  |  |  |
| Adjusted R^2^ | 0.267 |  |  |

**B1.** Predictors of 14-year change in area for MBHMS women

| **Multivariate Model** | **Estimate (95% CI)** | **Standardized β** | **p-value** |
| --- | --- | --- | --- |
| HT-use | 0.006 (-0.048, 0.060) | 0.009 | 0.832 |
| Baseline age | -0.002 (-0.006, 0.003) | -0.030 | 0.494 |
| **Baseline weight** | **-0.002 (-0.004, -0.001)** | **-0.151** | **0.010** |
| **Baseline height** | **0.018 (0.013, 0.022)** | **0.395** | **<0.001** |
| Baseline aBMD | -0.165 (-0.351, 0.021) | -0.085 | 0.082 |
| **Baseline area** | **-0.251 (-0.344, -0.159)** | **-0.314** | **<0.001** |
| Change in weight | 0.000 (-0.002, 0.003) | 0.016 | 0.715 |
|  |  |  |  |
| Adjusted R^2^ | 0.189 |  |  |

**B2.** Predictors of 14-year change in BMC for MBHMS women

| **Multivariate Model** | **Estimate (95% CI)** | **Standardized β** | **p-value** |
| --- | --- | --- | --- |
| **HT-use** | **0.143 (0.048, 0.239)** | **0.122** | **0.003** |
| **Baseline age** | **-0.024 (-0.033, -0.016)** | **-0.245** | **<0.001** |
| Baseline weight | -0.002 (-0.005, 0.001) | -0.078 | 0.168 |
| **Baseline height** | **0.010 (0.002, 0.017)** | **0.115** | **0.017** |
| **Baseline aBMD** | **-0.845 (-1.174, -0.516)** | **-0.237** | **<0.001** |
| **Baseline area** | **-0.243 (-0.406, -0.079)** | **-0.165** | **0.004** |
| **Change in weight** | **0.007 (0.004, 0.011)** | **0.163** | **<0.001** |
|  |  |  |  |
| Adjusted R^2^ | 0.253 |  |  |

**B3.** Predictors of 14-year change in aBMD for MBHMS women

| **Multivariate Model** | **Estimate (95% CI)** | **Standardized β** | **p-value** |
| --- | --- | --- | --- |
| **HT-use** | **0.027 (0.012, 0.042)** | **0.138** | **<0.001** |
| **Baseline age** | **-0.005 (-0.006, -0.004)** | **-0.309** | **<0.001** |
| **Baseline weight** | **0.001 (0.000, 0.001)** | **0.130** | **0.015** |
| Baseline height | 0.001 (-0.000, 0.002) | 0.076 | 0.093 |
| **Baseline aBMD** | **-0.239 (-0.292, -0.187)** | **-0.398** | **<0.001** |
| **Baseline area** | **-0.043 (-0.069, -0.017)** | **-0.172** | **0.001** |
| **Change in weight** | **0.002 (0.001, 0.002)** | **0.217** | **<0.001** |
|  |  |  |  |
| Adjusted R^2^ | 0.333 |  |  |

**C1.** Predictors of 10-year change in area for Health ABC-women

| **Multivariate Model** | **Estimate (95% CI)** | **Standardized β** | **p-value** |
| --- | --- | --- | --- |
| Race/ethnicity |  |  |  |
| **Black** | **-0.006 (-0.010, -0.001)** | **-0.101** | **0.010** |
| White | ref | ref | Ref |
| HT-use | 0.003 (-0.002, 0.007) | 0.043 | 0.236 |
| Baseline age | -0.0001 (-0.0008, 0.0006) | -0.011 | 0.759 |
| Baseline weight | 0.001 (-0.002, 0.003) | 0.025 | 0.576 |
| **Baseline height** | **0.005 (0.002, 0.007)** | **0.164** | **<0.001** |
| Baseline aBMD | -0.001 (-0.003, 0.002) | -0.028 | 0.530 |
| **Baseline area** | **-0.009 (-0.012, -0.007)** | **-0.332** | **<0.001** |
| Change in weight | -0.0003 (-0.002, 0.002) | -0.012 | 0.744 |
|  |  |  |  |
| Adjusted R^2^ | 0.087 |  |  |

**C2.** Predictors of 10-year change in BMC for Health ABC-women

| **Multivariate Model** | **Estimate (95% CI)** | **Standardized β** | **p-value** |
| --- | --- | --- | --- |
| Race/ethnicity |  |  |  |
| **Black** | **-0.010 (-0.016, -0.004)** | **-0.121** | **0.002** |
| White | ref | ref | ref |
| HT-use | 0.004 (-0.003, 0.010) | 0.040 | 0.255 |
| Baseline age | -0.0009 (-0.0018, 0.0001) | -0.061 | 0.071 |
| Baseline weight | 0.0004 (-0.003, 0.004) | 0.009 | 0.836 |
| **Baseline height** | **0.003 (0.0003, 0.006)** | **0.083** | **0.029** |
| **Baseline aBMD** | **-0.007 (-0.010, -0.003)** | **-0.170** | **<0.001** |
| **Baseline area** | **-0.008 (-0.011, -0.005)** | **-0.212** | **<0.001** |
| **Change in weight** | **0.009 (0.007, 0.012)** | **0.236** | **<0.001** |
|  |  |  |  |
| Adjusted R^2^ | 0.135 |  |  |

**C3.** Predictors of 10-year change in aBMD for Health ABC-women

| **Multivariate Model** | **Estimate (95% CI)** | **Standardized β** | **p-value** |
| --- | --- | --- | --- |
| Race/ethnicity |  |  |  |
| **Black** | **-0.001 (-0.002, -0.0003)** | **-0.093** | **0.013** |
| White | ref | Ref | ref |
| HT-use | 0.0004 (-0.0006, 0.0014) | 0.029 | 0.401 |
| **Baseline age** | **-0.0002 (-0.0003, -0.0000)** | **-0.066** | **0.047** |
| Baseline weight | 0.00004 (-0.0005, 0.0006) | 0.006 | 0.883 |
| Baseline height | 0.00005 (-0.0004, 0.0005) | 0.007 | 0.842 |
| **Baseline aBMD** | **-0.0013 (-0.0019, -0.0008)** | **-0.211** | **<0.001** |
| Baseline area | -0.0001 (-0.0005, 0.0004) | -0.013 | 0.714 |
| **Change in weight** | **0.0019 (0.0015, 0.0023)** | **0.304** | **<0.001** |
|  |  |  |  |
| Adjusted R^2^ | 0.175 |  |  |

**D1.** Predictors of 10-year change in area for Health ABC-men

| **Multivariate Model** | **Estimate (95% CI)** | **Standardized β** | **p-value** |
| --- | --- | --- | --- |
| Race/ethnicity |  |  |  |
| Black | -0.003 (-0.008, 0.002) | -0.050 | 0.201 |
| White | ref | ref | Ref |
| Baseline age | 0.0000 (-0.0008, 0.0008) | 0.002 | 0.953 |
| Baseline weight | -0.0005 (-0.003, 0.002) | -0.015 | 0.733 |
| Baseline height | 0.002 (-0.001, 0.004) | 0.059 | 0.198 |
| Baseline aBMD | -0.001 (-0.004, 0.001) | -0.042 | 0.319 |
| **Baseline area** | **-0.010 (-0.012, -0.007)** | **-0.326** | **<0.001** |
| Change in weight | 0.001 (-0.001, 0.003) | 0.030 | 0.424 |
|  |  |  |  |
| Adjusted R^2^ | 0.080 |  |  |

**D2.** Predictors of 10-year change in BMC for Health ABC-men

| **Multivariate Model** | **Estimate (95% CI)** | **Standardized β** | **p-value** |
| --- | --- | --- | --- |
| Race/ethnicity |  |  |  |
| **Black** | **-0.010 (-0.019, -0.002)** | **-0.096** | **0.012** |
| White | ref | ref | ref |
| Baseline age | -0.001 (-0.002, 0.0005) | -0.045 | 0.222 |
| Baseline weight | 0.002 (-0.003, 0.006) | 0.030 | 0.480 |
| Baseline height | -0.001 (-0.005, 0.004) | -0.012 | 0.783 |
| **Baseline aBMD** | **-0.005 (-0.009, -0.001)** | **-0.110** | **0.007** |
| **Baseline area** | **-0.013 (-0.017, -0.009)** | **-0.270** | **<0.001** |
| **Change in weight** | **0.012 (0.008, 0.016)** | **0.241** | **<0.001** |
|  |  |  |  |
| Adjusted R^2^ | 0.142 |  |  |

**D3.** Predictors of 10-year change in aBMD for Health ABC-men

| **Multivariate Model** | **Estimate (95% CI)** | **Standardized β** | **p-value** |
| --- | --- | --- | --- |
| Race/ethnicity |  |  |  |
| **Black** | **-0.001 (-0.002, -0. 00006)** | **-0.080** | **0.040** |
| White | ref | Ref | ref |
| Baseline age | -0.0001 (-0.0003, 0.0000) | -0.059 | 0.115 |
| Baseline weight | 0.0004 (-0.0002, 0.001) | 0.061 | 0.161 |
| Baseline height | -0.0004 (-0.001, 0.0002) | -0.061 | 0.178 |
| **Baseline aBMD** | **-0.0007 (-0.0013, -0.0002)** | **-0.105** | **0.013** |
| Baseline area | -0.0005 (-0.0011, 0.0001) | -0.074 | 0.087 |
| **Change in weight** | **0.0020 (0.0015, 0.0025)** | **0.282** | **<0.001** |
|  |  |  |  |
| Adjusted R^2^ | 0.108 |  |  |

**E1.** Predictors of 14-year change in area for MrOS men

| **Multivariate Model** | **Estimate (95% CI)** | **Standardized β** | **p-value** |
| --- | --- | --- | --- |
| Race/ethnicity |  |  |  |
| Non-White | -0.001 (-0.003, 0.002) | -0.010 | 0.658 |
| White | ref | ref | Ref |
| Baseline age | 0.0001 (-0.0001, 0.0002) | 0.014 | 0.541 |
| **Baseline weight** | **0.001 (0.0003, 0.002)** | **0.077** | **0.007** |
| **Baseline height** | **0.002 (0.001, 0.003)** | **0.130** | **<0.001** |
| **Baseline aBMD** | **0.001 (0.0002, 0.002)** | **0.060** | **0.016** |
| **Baseline area** | **-0.006 (-0.007, -0.005)** | **-0.358** | **<0.001** |
| Change in weight | -0.0005 (-0.001, 0.0003) | -0.028 | 0.238 |
|  |  |  |  |
| Adjusted R^2^ | 0.105 |  |  |

**E2.** Predictors of 14-year change in BMC for MrOS men

| **Multivariate Model** | **Estimate (95% CI)** | **Standardized β** | **p-value** |
| --- | --- | --- | --- |
| Race/ethnicity |  |  |  |
| Non-White | 0.004 (-0.001, 0.009) | 0.037 | 0.113 |
| White | ref | ref | ref |
| **Baseline age** | **-0.0005 (-0.0008, -0.0001)** | **-0.063** | **0.008** |
| **Baseline weight** | **0.004 (0.002, 0.006)** | **0.128** | **<0.001** |
| Baseline height | -0.0005 (-0.002, 0.001) | -0.015 | 0.607 |
| Baseline aBMD | -0.0005 (-0.002, 0.001) | -0.017 | 0.492 |
| **Baseline area** | **-0.005 (-0.007, -0.003)** | **-0.159** | **<0.001** |
| **Change in weight** | **0.008 (0.007, 0.010)** | **0.265** | **<0.001** |
|  |  |  |  |
| Adjusted R^2^ | 0.093 |  |  |

**E3.** Predictors of 14-year change in aBMD for MrOS men

| **Multivariate Model** | **Estimate (95% CI)** | **Standardized β** | **p-value** |
| --- | --- | --- | --- |
| Race/ethnicity |  |  |  |
| Non-White | 0.0006 (-0.0001, 0.0014) | 0.039 | 0.095 |
| White | ref | Ref | ref |
| **Baseline age** | **-0.0001 (-0.0002, -0.0000)** | **-0.087** | **<0.001** |
| **Baseline weight** | **0.0005 (0.0003, 0.0008)** | **0.109** | **<0.001** |
| **Baseline height** | **-0.0003 (-0.0006, -0.0000)** | **-0.065** | **0.028** |
| **Baseline aBMD** | **-0.0003 (-0.0005, -0.0001)** | **-0.061** | **0.014** |
| Baseline area | 0.0001 (-0.0001, 0.0004) | 0.024 | 0.395 |
| **Change in weight** | **0.0015 (0.0012, 0.0017)** | **0.309** | **<0.001** |
|  |  |  |  |
| Adjusted R^2^ | 0.102 |  |  |

**Table S2.** Characteristics for the five analytic cohorts (having baseline and last visit data) segregated into height-adjusted area tertiles. Differences in proportions of individuals by race/ethnicity sorted into the tertiles was determined by a Chi-squared goodness-of-fit test. Differences among groups were determined by ANOVA (overall p-value shown) with differences between groups determined by Tukey’s post hoc tests (1=different from narrow, 2=different from intermediate, 3=different from wide; p<0.05). Significant differences are shown in bold font.

**A. SWAN women**

|  | **Narrow**  **(n=436)** | **Intermediate**  **(n=435)** | **Wide**  **(n=436)** | **p-value** |
| --- | --- | --- | --- | --- |
| **Baseline Age (years)** | **46.0 ± 2.6^3^** | **46.2 ± 2.7** | **46.5 ± 2.5^1^** | **0.023** |
| Baseline Height (cm) | 162.8 ± 6.4 | 162.5 ± 6.7 | 162.7 ± 6.5 | 0.841 |
| **Baseline Weight (kg)** | **70.6 ± 17.3^2^** | **74.2 ± 19.1^1^** | **73.3 ± 18.2** | **0.011** |
| **Baseline aBMD (g/cm^2^)** | **0.86 ± 0.13^3^** | **0.86 ± 0.13^3^** | **0.83 ± 0.12^1,2^** | **<0.001** |
| **Baseline BMC (g)** | **3.82 ± 0.65^2,3^** | **4.08 ± 0.67^1,3^** | **4.23 ± 0.66^1,2^** | **<0.001** |
| **Baseline Area (cm^2^)** | **4.43 ± 0.28^2,3^** | **4.77 ± 0.21^1,3^** | **5.12 ± 0.27^1,2^** | **<0.001** |
| **Race/ethnicity (n, %)** |  |  |  | **<0.001** |
| **Black** | **138 (38.3)** | **124 (34.4)** | **98 (27.2)** | **0.032** |
| **White** | **184 (28.6)** | **218 (33.9)** | **241 (37.5)** | **0.022** |
| **Chinese** | **83 (53.2)** | **48 (30.8)** | **25 (16.0)** | **<0.001** |
| **Japanese** | **31 (20.9)** | **45 (30.4)** | **72 (48.6)** | **<0.001** |
| HT use (n, %) | 2 (0.5) | 0 (0.0) | 0 (0.0) | 0.333 |

**B. MBHMS women**

|  | **Narrow**  **(n=146)** | **Intermediate**  **(n=145)** | **Wide**  **(n=146)** | **p-value** |
| --- | --- | --- | --- | --- |
| Baseline Age (years) | **39.0 ± 4.9^3^** | **40.3 ± 5.0** | **40.9 ± 5.0^1^** | **0.004** |
| Baseline Height (cm) | 163.6 ± 6.4 | 162.6 ± 5.6 | 163.8 ± 6.0 | 0.219 |
| Baseline Weight (kg) | **65.2 ± 11.4^2, 3^** | **71.7 ± 15.3^1, 3^** | **84.4 ± 19.1^1, 2^** | **<0.001** |
| Baseline aBMD (g/cm^2^) | 0.98 ± 0.13 | 1.00 ± 0.14 | 1.01 ± 0.15 | 0.209 |
| Baseline BMC (g) | **4.39 ± 0.69^2, 3^** | **4.78 ± 0.75^1, 3^** | **5.07 ± 0.85^1, 2^** | **<0.001** |
| Baseline Area (cm^2^) | **4.25 ± 0.21^2, 3^** | **4.55 ± 0.17^1, 3^** | **4.90 ± 0.24^1, 2^** | **<0.001** |
| Race/ethnicity (n, %) |  |  |  |  |
| Black |  |  |  |  |
| White | 146 (100) | 145 (100) | 146 (100) |  |
| Chinese |  |  |  |  |
| Japanese |  |  |  |  |
| HT use (n, %) | 33 (22.6) | 35 (24.1) | 35 (24.0) | 0.959 |

**C. Health ABC-women**

|  | **Narrow**  **(n=261)** | **Intermediate**  **(n=265)** | **Wide**  **(n=260)** | **p-value** |
| --- | --- | --- | --- | --- |
| Baseline Age (years) | 72.9 ± 2.9 | 73.2 ± 2.7 | 73.1 ± 2.8 | 0.295 |
| Baseline Height (cm) | 160.1 ± 6.5 | 159.1 ± 6.2 | 159.9 ± 5.9 | 0.120 |
| Baseline Weight (kg) | 70.1 ± 14.1 | 68.6 ± 13.0 | 70.9 ± 14.3 | 0.160 |
| **Baseline aBMD (g/cm^2^)** | **0.73 ± 0.12^2,3^** | **0.67 ± 0.11^1^** | **0.67 ± 0.13^1^** | **<0.001** |
| **Baseline BMC (g)** | **3.21 ± 0.64^3^** | **3.32 ± 0.57^3^** | **3.59 ± 0.74^1,2^** | **<0.001** |
| **Baseline Area (cm^2^)** | **4.40 ± 0.47^2,3^** | **4.92 ± 0.21^1,3^** | **5.33 ± 0.28^1,2^** | **<0.001** |
| **Race/ethnicity (n, %)** |  |  |  | **<0.001** |
| **Black** | **129 (43.0)** | **85 (28.3)** | **86 (28.7)** | **0.018** |
| **White** | **132 (27.2)** | **180 (37.0)** | **174 (35.8)** | **0.002** |
| HT use (n, %) | 53 (27.3) | 70 (36.1) | 71 (36.6) | 0.064 |

**D. Health ABC-men**

|  | **Narrow**  **(n=228)** | **Intermediate**  **(n=230)** | **Wide**  **(n=226)** | **p-value** |
| --- | --- | --- | --- | --- |
| **Baseline Age (years)** | **73.2 ± 2.7^3^** | **73.1 ± 2.7^3^** | **73.8 ± 2.8^1,2^** | **0.008** |
| Baseline Height (cm) | 173.1 ± 7.0 | 174.2 ± 6.5 | 173.4 ± 6.7 | 0.234 |
| Baseline Weight (kg) | 81.4 ± 14.0 | 82.7 ± 12.2 | 81.7 ± 12.8 | 0.533 |
| **Baseline aBMD (g/cm^2^)** | **0.84 ±0.13^2,3^** | **0.79 ± 0.12^1,3^** | **0.76 ± 0.13^1,2^** | **<0.001** |
| **Baseline BMC (g)** | **4.38 ± 0.77^2,3^** | **4.58 ± 0.76^1^** | **4.69 ± 0.90^1^** | **<0.001** |
| **Baseline Area (cm^2^)** | **5.23 ± 0.46^2,3^** | **5.75 ± 0.24^1,3^** | **6.15 ± 0.33^1,2^** | **<0.001** |
| **Race/ethnicity (n, %)** |  |  |  | **<0.001** |
| **Black** | **100 (50.0)** | **63 (31.5)** | **37 (18.5)** | **<0.001** |
| **White** | **128 (26.4)** | **167 (34.5)** | **189 (39.1)** | **0.002** |

**E. MrOS**

|  | **Narrow**  **(n=584)** | **Intermediate**  **(n=573)** | **Wide**  **(n=564)** | **p-value** |
| --- | --- | --- | --- | --- |
| Baseline Age (years) | 70.2 ± 4.1 | 70.2 ± 4.0 | 70.6 ± 4.3 | 0.127 |
| Baseline Height (cm) | 175.6 ± 6.7 | 175.0 ± 6.6 | 175.2 ± 6.7 | 0.320 |
| Baseline Weight (kg) | 83.4 ± 11.9 | 84.2 ± 13.1 | 84.3 ± 11.8 | 0.430 |
| **Baseline aBMD (g/cm^2^)** | **0.82 ±0.13^2,3^** | **0.79 ± 0.12^1,3^** | **0.77 ± 0.11^1,2^** | **<0.001** |
| **Baseline BMC (g)** | **4.38 ± 0.72^2,3^** | **4.50 ± 0.70^1,3^** | **4.68 ± 0.73^1,2^** | **<0.001** |
| **Baseline Area (cm^2^)** | **5.31 ± 0.30^2,3^** | **5.68 ± 0.24^1,3^** | **6.07 ± 0.31^1,2^** | **<0.001** |
| **Race/ethnicity (n, %)** |  |  |  |  |
| **Black** | **35 (67.3)** | **14 (26.9)** | **3 (5.8)** | **<0.001** |
| White | 505 (32.5) | 516 (33.2) | 534 (34.3) | 0.343 |
| Asian | 23 (42.6) | 15 (27.8) | 16 (29.6) | 0.402 |
| **Other** | **21 (35.0)** | **28 (46.7)** | **11 (18.3)** | **0.029** |

**Table S3.** Characteristics for the five analytic cohorts (having baseline and last visit data) segregated into baseline femoral neck aBMD tertiles. Differences in proportions of individuals by race/ethnicity sorted into the tertiles was determined by a Chi-squared goodness-of-fit test. Differences among groups were determined by ANOVA (overall p-value shown) with differences between groups determined by Tukey’s post hoc tests (1=different from narrow, 2=different from intermediate, 3=different from wide; p<0.05). Significant differences are shown in bold font.

**A. SWAN women**

|  | **Low**  **(n=437)** | **Intermediate**  **(n=434)** | **High**  **(n=436)** | **p-value** |
| --- | --- | --- | --- | --- |
| Baseline Age (years) | 46.4 ± 2.7 | 46.2 ± 2.6 | 46.1 ± 2.6 | 0.227 |
| Baseline Height (cm) | **160.8 ± 6.5^2, 3^** | **162.8 ± 6.3^1, 3^** | **164.4 ± 6.4^1, 2^** | **<0.001** |
| Baseline Weight (kg) | **61.0 ± 11.5^2, 3^** | **71.5 ± 15.0^1, 3^** | **85.6 ± 18.4^1, 2^** | **<0.001** |
| Baseline aBMD (g/cm^2^) | **0.72 ± 0.05^2, 3^** | **0.84 ± 0.03^1, 3^** | **0.99 ± 0.08^1, 2^** | **<0.001** |
| Baseline BMC (g) | **3.41 ± 0.37^2, 3^** | **3.99 ± 0.35^1, 3^** | **4.73 ± 0.52^1, 2^** | **<0.001** |
| Baseline Area (cm^2^) | 4.77 ± 0.40 | 4.78 ± 0.37 | 4.78 ± 0.37 | 0.865 |
| Race/ethnicity (n, %) |  |  |  | **<0.001** |
| Black | 35 (9.7) | 104 (28.9) | 221 (61.4) | **<0.001** |
| White | 232 (36.1) | 228 (35.5) | 183 (28.5) | **0.032** |
| Chinese | 83 (53.2) | 54 (34.6) | 19 (12.2) | **<0.001** |
| Japanese | 87 (58.8) | 48 (32.4) | 13 (8.8) | **<0.001** |
| HT use (n, %) | 0 (0.0) | 0 (0.0) | 2 (0.5) | 0.221 |

**B. MBHMS women**

|  | **Low**  **(n=147)** | **Intermediate**  **(n=144)** | **High**  **(n=146)** | **p-value** |
| --- | --- | --- | --- | --- |
| Baseline Age (years) | 40.2 ± 4.9 | 40.2 ± 5.0 | 39.8 ± 5.2 | 0.789 |
| Baseline Height (cm) | **161.8 ± 5.9^2, 3^** | **163.8 ± 6.4^1^** | **164.4 ± 5.4^1^** | **<0.001** |
| Baseline Weight (kg) | **66.8 ± 13.2^2, 3^** | **71.5 ± 15.7^1, 3^** | **83.1 ± 18.9^1, 2^** | **<0.001** |
| Baseline aBMD (g/cm^2^) | **0.85 ± 0.07^2, 3^** | **0.99 ± 0.03^1, 3^** | **1.15 ± 0.08^1, 2^** | **<0.001** |
| Baseline BMC (g) | **4.01 ± 0.51^2, 3^** | **4.71 ± 0.43^1, 3^** | **5.54 ± 0.59^1, 2^** | **<0.001** |
| Baseline Area (cm^2^) | **4.52 ± 0.36^3^** | **4.56 ± 0.33** | **4.62 ± 0.32^1^** | **0.026** |
| Race/ethnicity (n, %) |  |  |  |  |
| Black |  |  |  |  |
| White | 147 (100) | 144 (100) | 146 (100) |  |
| Chinese |  |  |  |  |
| Japanese |  |  |  |  |
| HT use (n, %) | 40 (27.2) | 29 (20.1) | 34 (23.3) | 0.370 |

**C. Health ABC-women**

|  | **Low**  **(n=264)** | **Medium**  **(n=265)** | **High**  **(n=257)** | **p-value** |
| --- | --- | --- | --- | --- |
| Baseline Age (years) | 73.3 ± 2.8 | 73.1 ± 2.8 | 72.8 ± 2.7 | 0.071 |
| **Baseline Height (cm)** | **158.4 ± 6.0^2,3^** | **160.0 ± 6.2^1^** | **160.7 ± 6.2^1^** | **<0.001** |
| **Baseline Weight (kg)** | **61.9 ± 10.4^2,3^** | **69.6 ± 11.7^1,3^** | **78.4 ± 13.9^1,2^** | **<0.001** |
| **Baseline aBMD (g/cm^2^)** | **0.56 ± 0.05^2,3^** | **0.68 ± 0.03^1,3^** | **0.83 ± 0.09^1,2^** | **<0.001** |
| **Baseline BMC (g)** | **2.80 ± 0.35^2,3^** | **3.34 ± 0.37^1,3^** | **3.99 ± 0.63^1,2^** | **<0.001** |
| **Baseline Area (cm^2^)** | **4.97 ± 0.46^3^** | **4.89 ± 0.48** | **4.79 ± 0.57^1^** | **<0.001** |
| **Race/ethnicity (n, %)** |  |  |  | **<0.001** |
| **Black** | **47 (15.7)** | **95 (31.7)** | **158 (52.7)** |  |
| **White** | **217 (44.6)** | **170 (35.0)** | **99 (20.4)** |  |
| HT use (n, %) | 57 (29.4) | 71 (36.6) | 66 (34.0) | 0.276 |

**D. Health ABC-men**

|  | **Low**  **(n=226)** | **Medium**  **(n=230)** | **High**  **(n=228)** | **p-value** |
| --- | --- | --- | --- | --- |
| **Baseline Age (years)** | **73.9 ± 2.9^2,3^** | **73.2 ± 2.8^1^** | **73.0 ± 2.6^1^** | **0.001** |
| **Baseline Height (cm)** | **171.5 ± 6.4^2,3^** | **174.2 ± 6.8^1^** | **175.0 ± 6.6^1^** | **<0.001** |
| **Baseline Weight (kg)** | **76.5 ± 11.1^2,3^** | **82.1 ± 11.4^1,3^** | **87.2 ± 14.1^1,2^** | **<0.001** |
| **Baseline aBMD (g/cm^2^)** | **0.66 ±0.05^2,3^** | **0.79 ± 0.03^1,3^** | **0.94 ± 0.09^1,2^** | **<0.001** |
| **Baseline BMC (g)** | **3.82 ± 0.41^2,3^** | **4.50 ± 0.45^1,3^** | **5.32 ± 0.72^1,2^** | **<0.001** |
| **Baseline Area (cm^2^)** | **5.78 ± 0.41^3^** | **5.70 ± 0.53** | **5.64 ± 0.59^1^** | **0.014** |
| **Race/ethnicity (n, %)** |  |  |  | **<0.001** |
| **Black** | **29 (14.5)** | **73 (36.5)** | **98 (49.0)** |  |
| **White** | **197 (40.7)** | **157 (32.4)** | **130 (26.9)** |  |

**E. MrOS**

|  | **Low**  **(n=581)** | **Medium**  **(n=577)** | **High**  **(n=563)** | **p-value** |
| --- | --- | --- | --- | --- |
| **Baseline Age (years)** | **70.8 ± 4.5^3^** | **70.5 ± 4.1^3^** | **69.8 ± 3.9^1,2^** | **<0.001** |
| **Baseline Height (cm)** | **174.7 ± 6.4** | **175.5 ± 6.8** | **175.6 ± 6.8** | **0.031** |
| **Baseline Weight (kg)** | **79.7 ± 11.1^2,3^** | **84.5 ± 11.4^1,3^** | **87.9 ± 12.9^1,2^** | **<0.001** |
| **Baseline aBMD (g/cm^2^)** | **0.67 ±0.05^2,3^** | **0.79 ± 0.03^1,3^** | **0.93 ± 0.09^1,2^** | **<0.001** |
| **Baseline BMC (g)** | **3.87 ± 0.40^2,3^** | **4.47 ± 0.38^1,3^** | **5.23 ± 0.60^1,2^** | **<0.001** |
| **Baseline Area (cm^2^)** | **5.75 ± 0.41^2,3^** | **5.68 ± 0.44^1,3^** | **5.62 ± 0.40^1,2^** | **<0.001** |
| **Race/ethnicity (n, %)** |  |  |  | **0.027** |
| **Black** | **5 (9.6)** | **19 (36.5)** | **28 (53.9)** |  |
| White | 543 (34.9) | 519 (33.4) | 493 (31.7) |  |
| Asian | 14 (25.9) | 23 (42.6) | 17 (31.5) |  |
| Other | 19 (31.7) | 16 (26.7) | 25 (41.7) |  |
